# An Updated Review on Efficiency of *Penthorum chinense* Pursh in Traditional Uses, Toxicology, and Clinical Trials

**DOI:** 10.1155/2023/4254051

**Published:** 2023-02-18

**Authors:** Fazul Nabi, Jameel Ahmed, Weilai Tao, Qin Lu, Zohaib Ahmed Bhutto, Ayaz Qadir, Abdul Hameed, Iftikhar Ahmed, Nazeer Ahmed, Muhammad Essa, Juan Liu

**Affiliations:** ^1^Department of Traditional Chinese Veterinary Medicine, College of Veterinary Medicine, Southwest University, Chongqing, China; ^2^Faculty of Veterinary and Animal Science, Lasbela University of Agriculture, Water and Marine Sciences, Uthal, Pakistan; ^3^Department of Economics, Faculty of Management and Social Science, Lasbela University of Agriculture, Water and Marine Sciences, Uthal, Pakistan; ^4^King Edward Medical University, Lahore Punjab, Pakistan; ^5^Chinese Veterinary Herbal Drugs Innovation Research Laboratory, University Veterinary Science Engineering Research Center in Chongqing, Chongqing, China

## Abstract

Traditional Chinese medicines (TCM) play an important role in the control and treatment of several animal diseases. Penthorum chinense Pursh (PCP) is a famous plant for its use in traditional medication practice and therapeutic effects in numerous pathological conditions. In China, PCP is utilized for both food and medication due to numerous bioactivities. PCP is widely administered in prevention and treatment of traumatic injury, edema, and liver diseases with functions of reducing swelling, support diuresis, blood stasis, and mitigation symptoms of excessive alcohol intake. Recently, PCP highlighted for research trials in various fields including pharmacology, pharmacognosy, cosmeceuticals, nutraceuticals, and pharmaceuticals due to medicinal significance with less toxicity and an effective ethnomedicine in veterinary practice. PCP contains diverse important ingredients such as flavonoids, organic acids, coumarins, lignans, polyphenols, and sterols that are important bioactive constituents of PCP exerting the therapeutic benefits and organ-protecting effects. In veterinary, PCP extract, compound, and phytochemicals/biomolecules significantly reversed the liver and kidney injuries, via antioxidation, oxidative stress, apoptosis, mitochondrial signaling pathways, and related genes. PCP water extract and compounds also proved in animal and humans' clinical trial for their hepatoprotective, antiaging, nephroprotective, anti-inflammatory, antidiabetic, antibacterial, antiapoptotic, immune regulation, and antioxidative stress pathways. This updated review spotlighted the current information on efficiency and application of PCP by compiling and reviewing recent publications on animal research. In addition, this review discussed the toxicology, traditional use, comparative, and clinical application of PCP in veterinary practices to authenticate and find out new perspectives on the research and development of this herbal medicine.

## 1. Introduction

In recent hundred years, researchers focus in on Penthorum chinense Pursh (PCP), and PCP is a perennial herb, Chinese traditional medicine used for its hepatoprotective effects such as liver edema, infectious hepatitis, and liver injuries [1, 2]. It is well established that PCP extract and PCP biomolecules have a range of pharmacological and health-promoting benefits, including anti-inflammatory, hepatoprotective, and antioxidant properties [3–5]. Hepatoprotective effect of PCP is due to cure liver damage by keeping oxygen-free radicals out and reducing inflammatory response [6–9]. These pharmacological effects result from the PCPE's important bioactive constituents, such as the flavonoids (quercetin, 5-hydroxy-flavanone-7-O-D-glucoside, and kaempferol), lignans, coumarins, steroids, polyphenols, terpenoids, pinocembrin, catechins, and organic acids [10].

Various researches have shown that many extracts from PCP have significant pharmacological activities of antioxidation and anti-inflammation [3–5]. PCP is a traditional medication that has a high concentration of medicinal ingredients such organic acids, flavonoids, and terpenoids. It is mostly used to treat liver illness and has excellent curative and little harmful effects). According to some studies, Penthorum chinense Purse extracts contain the compounds quercetin, pinocembrin, catechins, 5-hydroxy-flavanone-7-O-D-glucoside, and kaempferol. It is proved with infrared spectroscopy that flavonoids (kaempferol, quercetin, and pinocembrin-7-O-beta-D-glucoside) are dominant biomolecules found in PCPE, which produced liver-protective effects, antioxidation, and anti-inflammation [11, 12].

PCPEs primarily consist of flavonoids. We also confirmed via infrared spectroscopy that PCPE included a high concentration of flavonoids and polyphenols, which may be responsible for the hepatoprotective effect. For instance, it has been noted that many flavonoids and phenolic compounds extracted from PCP have potent antioxidant and anti-inflammatory properties as well as significant liver-protective effects. These compounds include kaempferol, quercetin, and pinocembrin-7-O-beta-D-glucoside; in China, 2000 years ago, TCM is used to treat a variety of diseases in human clinical practices, and recently, TCM draw more attention and popularity due to their successful and safe experimental clinical trial [13].

The domains of life sciences have been modernized by the application of integrated pharmacology and biological networks. The technique of multiple target control is used to predict the primary active ingredient and potential target populations of traditional Chinese medicine and to develop the mechanism by which TCMs exert curative effects. It is based on the construction of drug-target networks and analysis of network characteristics [14, 15]. However, the main barrier to TCM's acceptability globally is the inability to clearly explain its application method due to its complicated components. Because the primary components are the chemical substances that produce the medication effects, a study of the chemical components in TCM will help to clarify the mechanism. Renewed studies gained interest in PCP research and greatly increased potential use of PCP against liver diseases, but varieties of animal models, disease-induced models, cross studies, and dosage use of PCP rigorous demand to reach the existing findings. Furthermore, studies focused on pharmacology and chemical constituents only found the activities and effects of the active ingredients of PCP such as polyphenols and other compounds and their mechanism of actions. This updated review links a bridge between the previous and recent pharmacological studies of PCP and a revised summary of current progress in the aspect of pharmacology, toxicology, and clinical trials, which highlights the evidence for use of PCP and points out the scientific gaps in the future research.

## 2. Medical Resource and Chemical Constituents

Most flavonoids have been found such as pinocembrin, quercetin, and kaempferol derivatives and isomers. 2,3′-Dihydroxy-3-methoxy-6′-methanone-benzophenone-4-O-glucoside and 2,4-dihydroxy-3-methoxy-6′-methanone-benzo-phenone-3′-O-glucoside are two of the four phenylpropanoids [16, 17]. Quercetin, the primary active component of PCP found to be useful for treating alcoholic liver injury, was discovered in the PCP aqueous extract [18].

**Figure 1:**
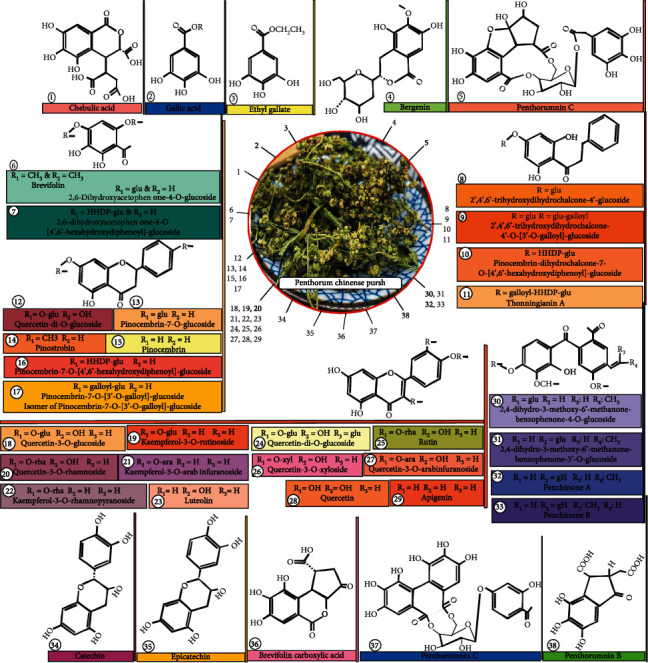
Dioramic representation of different compounds, their derivate, and isomers present in PCP.

The structural formula of 38 active compounds, isomers, and derivatives is given in Figure 1 including chebulic acid, gallic acid, ethyl gallate, bergenin, penthorummin C, 2,6-dihydroxyacetophen one-4-O-glucoside, 2,6-dihydroxyacetophen one-O[4′,6′-hexahydroxydiphenoly]-glucoside, quercetin-di-O-glucoside, pinocembrin-7-O-glucoside, pinostrobin, pinocembrin, pinocembrin-7-O-[4′,6′-hexahydroxydiphenoyl]-glucoside, pinocembrin-7-O-[3′-O-galloyl]-glucoside, isomer of pinocembrin-7-O-[3′-O-galloyl]-glucoside, quercetin-3-O-glucoside, kaempferol-3-O-rutinoside, quercetin-3-O-rhamnoside, kaempferol-3-O-arab infuranoside, kaempferol-3-O-rhamnopyranoside, luteolin, quercetin-di-O-glucoside, rutin, quercetin-3-xyloside, quercetin-3-O-arabinofuranoside, quercetin, apigenin, 2,4-dihydro-3-methoxy-6′-methanone-benzophenone-4-O-glucoside, 2,4-dihydro-3-methoxy-6′-methanone-benzophenone-3′-O-glucoside, penchinone A, penchinone B, catechin, epicatechin, brevifolin carboxylic acid, penthorumnin C, and penthorumnin B, with molecular formula, i.e., C_14_H_13_O_11_, C_7_H_7_O_5_, C_9_H11O5, C_14_H_17_O_9_, C_26_H_25_O_17_, C_14_H_19_O_9_, C_21_H_23_O_9_, C_16_H_15_O_4_, C_35_H_29_O_17_, C_28_H_27_O_13_, C_28_H_27_O_13_, C_21_H_21_O_12_, C_27_H_31_O_15_, C_21_H_21_O_11_, C_20_H_19_O_10_, C_21_H_21_O_10_, C_15_H_11_O_6_, C_27_H_31_O_17_, C_27_H_31_O_16_, C_20_H_19_O_11_, C_20_H_19_O_11_, C_15_H_11_O_7_, C_15_H_11_O_5_, C_25_H_29_O_11_, C_25_H_27_O_11_, C_19_H_19_O_6_, C_19_H_19_O_6_, C_15_H_15_O_6_, C_15_H_15_O_6_, C_13_H_9_O_8_, C_27_H_25_O_17_, and C_12_H_11_O_8_, respectively [9, 16, 17, 19, 20].

Recently, there are new phenolic compound, flavonoids, and neolignans, namely, (4′E)-2,3′-dihydroxy-3-methoxy-6′-methanone-benzophenone-4-O*β*-D-glucopyranoside, (4′E)-2,4-dihydroxy-3-methoxy-6′-methanone-benzophenone-3′-O*β*-D-glucopyranoside [21], 20,60-dihydroxydihydrochalcone-40-O-[200-O-galloyl 400,600-hexahydroxydiphenoyl]-b-D-glucopyranoside, pinocembrin-7-O-[300-O-galloyl]-b-D-glucose, pinocembrin-7-O-[200-O-galloyl-400,600-hexahydroxydiphenoyl]-b-D-glucose [9], (70 Z,8S)-3,8-dihydroxy-4-methoxy-2,40-epoxy-8,50-neolign-70-en-7-one-20-O-b-D-glucopyranose, and (70 Z,8S)-4,8-dihydroxy3-methoxy-2,40-epoxy-8,50-neolign-70-en-7-one-20-O-b-D-glucopyranose [22], which have protective effect on the liver and are helpful in free radical scavenging activities, antihyperlipidemic activities, and antiproliferative on hepatic stellate T6 cells (HSC-T6 cells), respectively.

## 3. Clinical and Comparative Activities

### 3.1. Antioxidation

Generally, ROS production is involved in regular cell metabolism, and it is generally compensated through antioxidant defense system to balance the specific redox stability [23]. Oxidative stress is a biological condition in which free radicals across the antioxidant capabilities, and oxidative stress is identified as major element in several diseases such as chronic liver diseases, such as hepatitis and alcoholic and nonalcoholic fatty liver diseases [24]. The liver is a vital organ with metabolic activities and normal physiological functions. The functions of the liver are correlated with gastrointestinal tract (GIT), and the disorder between this balance may danger to drug toxicity and introduction of xenobiotics within the organism [25, 26]. Therefore, the antioxidant treatment is very effective for the controlling the oxidative stress conditions and correct or control the equilibrium between the antioxidant and oxidants in the encouragement of the liver pathogenesis and protection of hepatocytes from excessive exposure to oxidative stress.

PCP-rich source of natural antioxidants is recently the focus of pharmaceutical, cosmaceutical, nutraceutical, and food industries [27]. Polyphenols demonstrated potent hepatoprotective benefits against oxidative injury by directly scavenging ROS and lowering liver enzymes as well as indirectly increasing antioxidant levels [12]. It was proved that activation of Nrf2 signaling pathway by PCPE produced hepatoprotective effect in CCl4-induced oxidative stress model [28].

Flavonoids upregulated the activities of superoxide dismutase and catalase while downregulating the level of malondialdehyde [29]. PCP and polysaccharide fraction PCPP-1a possessed strong hydroxyl radical scavenging activity, Fe^2+^ chelating, and DPPH radical scavenging [30, 31].

Pinocembrin decreases the oxidation by increasing scavenging of the free radical and antisuperoxide formation when taken in 30 *μ*M in rat [32] while increasing the cellular antioxidant defense and metal ion chelation [33]. Quercetin with different concentrations (10, 50, and 100 *μ*M) in rat resulted in decreases of H_2_O_2_ stress, ROS production, and ER stress [34, 35]. Kaempferol downregulated the production of ROS, mitochondrial membrane potential, and intracellular and intracellular ROS production [36, 37], due to scavenging ROS and activating the Nrf2-antioxidant signaling pathway. Polyphenols from PCP demonstrated a potent protective effect against high glucose- (HG-) associated vascular inflammation [3–5].

**Figure 2:**
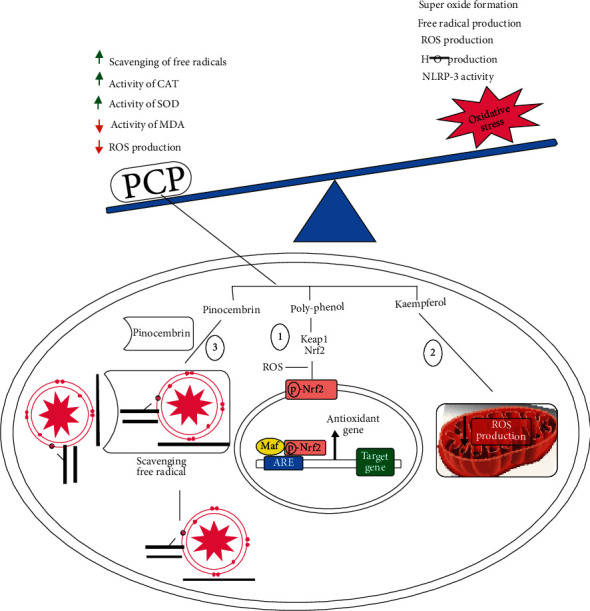
Diagram representation of oxidative stress production and protective effects of PCP. (1) Polyphenol reduces the ROS production by Nrf2 pathway. (2) Kaempferol downregulates ROS production in mitochondria. (3) Pinocembrin reduces the oxidative stress by scavenging free radicals

Thonningianin A (TA), a substance derived from PCP, effectively decreased the quantity of ROS in human umbilical vein endothelial cells that are stimulated by H_2_O_2_ HUVECs. Additionally, following the administration of TA, the expression of pro- and cleaved-IL-1 in the aortic artery of ApoE-KO mice was also reduced at the transcriptional and posttranscriptional levels, which may be related to the decrease in oxidative stress-related Nod-like receptor protein 3 (NLRP3) in the aortic arteries of ApoE-KO mice [5]. The structure of ThA is a combination of gallic acid esters of glucose and dihydrochalcone. The antioxidant properties of ThA have been previously reported [32]. Synthetic antioxidant (tannic acid) and ThA found similar activities; however, ThA is more effective than gallic acid, vitamins E and C in LPO-induced, and the deoxyribose assay. The properties of ThA are important for the free radial-mediated disease inhibition, and further studies are needed to explore the in vitro and in vivo experiments.  Figure 2 represents the oxidative stress production and protective effect of PCP.

### 3.2. Anti-Inflammatory

Inflammation process is well-known process to promote the pathological and physiological pathways by activating the various other systems such as the immune system, vascular system, and other cells within the damaged tissues [38]. The acute and chronic inflammation is caused by the several factors including some microorganism infection, chemical, surgical, and physical irritation. The classic type of inflammations is heat, edema, pain swelling, and redness [39]. Chronic inflammation or prolonged inflammation may also affects many other organs systems such as the heart, lungs, brain, and skin [40]; however, the chronic inflammation also connected with various pathogenesis and tissues damages can cause the serious cellar injury and variety of disease conditions such as Alzheimer's disease, diabetes, and carcinogenesis [41].

Penthorum chinense Pursh shows anti-inflammatory capabilities due to the presence of several polyphenol compounds that inhabits inflammation by activating the nuclear erythroid 2-related factor 2 in the liver of mice while downregulation of heme oxygenase^−1^ (Nrf2/HO^−1^) signaling pathway in the hepatocytes of human beings [42]. Other compound present in PCP decreases the amount of proprotein convertase subtilisin/kexin type 9 (PCSK9) and activates the low-density lipoprotein receptor (LDLR) in the hepatics cells [43]. It protects cell by anti-inflammatory mechanism like increasing the expression of anti-inflammatory cytokines interleukin-10 and TGF-*β* in fish intestine cells [44]. Similarly, PCP shows anti-inflammatory effect by downregulating mitogen-activated protein kinase (MAPK) and nuclear factor *κ*B (NF-*κ*B) signaling pathways [45]. PCP and its related compounds proved for anti-inflammatory effects could reverse inflammation of kidney, liver, and nervous tissues by interacting with discussed signaling pathways. Recent studies have also shown that PCP downregulated the overexpression of inflammatory cytokines and mediators in LPS-induced inflammatory response in animals and significantly inhibited the expression level of NO, TNF-*α*, and IL-1*β*, and it shows the significant anti-inflammatory effect together inhibiting the MyD88/TLR4/NF-*κ*B signaling pathways [46, 47]. Tao et al. studied the hepatoprotective effect of the PCPE in CCl4-induced liver injury in dogs and found that the NF-*κ*B and MAPK signaling pathways in dogs are associated to the antioxidant and anti-inflammatory effects of PCPE. Inflammation that occurred in liver tissues induced by CCl4 has been controlled through the administrations of PCPE in dogs, and inflammatory and proinflammatory factors (IL-1*β*, IL-6, and TNF) are the important mediators, involved in the response of inflammation, and anti-inflammatory factor such as IL-10 turns as an antagonistic inflammatory mediator pathways controlled in acute liver injury in dogs [45].

### 3.3. Antitoxic

In the view of the toxic effects of synthetic drugs, collected data resulted that existing treatment options have been limited the therapeutic success in animals and humans. Considerably over the last decade, the therapeutic use of herbal/plant medicines has been increased over the world. Recent severe complications and new disease with no treatment has promoted the belief that natural medicines are safe and less toxic. PCP is a nontoxic herbal medicine, and well-established reports indicated that PCP singly or with combined therapy is proved to be safest herbal medicine with no or negligible toxicity or side effects whether administered in dose/or time-dependent way [1]. It is reported that PCP induced cytotoxic effect possibly due to accumulation of reactive oxygen species (ROS) [2], while the extract of bioactive parts of PCP like stem, leaves, and flowers is used to protect cells from oxidants toxic damages as induced with H_2_O_2_ [48] [12]. The flavonoid contents from leafs of PCP protected the liver cells from lipotoxicity injury [1]. The toxic compound produced by several bacterial is also regularized by extract fraction of PCP like PGF and other components, i.e., pinocembrin-7-O-[4^″^,6^″^-hexahydroxydiphenoyl]-*β*-D-glucose, thonningianin A, and pinocembrin-7-O-[3^″^-O-galloyl-4′,6′-(s)-HHDP]-*β*-D-glucose (PGHG), that reduce the activity of methicillin resistance S. aureus (MRSA) [49]. The toxicity produced by aflatoxin *β*1 is one of the major toxicities in broiler chicken; this toxicity can be prevented by the use of PCPC as a natural and safe agent with supplemented in the diet [50]. PCP and its compounds could be included in the human and veterinary diet due to the best detoxification properties.

Pinocembrin, the primary flavonoid isolated from PCP, can be used as neuroprotective agent in cerebral ischemic injury along with the pharmacological effects almost in various systems. This flavonoid attracted recent interest due to the antitoxic effects [6, 51]. Quercetin is one of the polyphenolic compounds isolated from PCP and widely studied for anti-inflammatory and antioxidant compound. Quercetin prevents alcohol-induced hepatotoxicity [52], has scavenging properties [53], decreased the cytotoxicity [54], inhibits neurotoxicity [55], and possesses several medicinal benefits. Herbal antioxidants are major supporting agents in the battle of diseases and infections. There is a wide published data regarding PCP, their extract, or compound, there is no published information on their toxicity, and importantly, most of published data is available on their beneficial, scavenging, and biological effect when administered traditionally in a mixture or single form in animals. It is crucial to evaluate the advantageous application of these herbs when used in a combination or single according to a precisely defined recipe.

### 3.4. Antimicrobial

Flavonoids present in PCP showed inhibitory effects on many strains of bacteria including Escherichia coli, Pseudomonas aeruginosa, and Cryptococcus neoformans by destroying the microbial membrane, inhibiting the invasion of bacteria into the host cell, increasing the likelihood of bacterial apoptosis, and ceasing the bacterial fatty acid synthesis [56].

Flavonoids present in PCP are proved for antiviral capability [44, 57, 58], so PCP could be included in medicinal preparation of acute liver disease (acute viral hepatitis, chronic active viral hepatitis, and hepatitis B virus) [6, 28, 59] interfering with viral replication [1, 60]. Similarly, gallic acid also protects the liver from viral infections [61]; similarly, flavonoids also affected the indices of TNF-*α*, IL-6, and IL-1*β* in infection [56].

The flavonoid reported for antiviral effects against influenza A virus by an interaction with various mechanisms increases the expression of PKC*α* (protein kinase C alpha), VIPR1 (vasoactive intestinal polypeptide receptor 1), retinoic acid-inducible gene (RIG)-1, TRAF6 (TNF receptor associated factor 6), and Toll-like receptor (TLR)-7 [28] and by regulating the eTLR7, RIG-1, and AQP5 signaling pathways [56]. Furthermore, biomolecules of PCP can be investigated for their interactions with various signaling pathways and in various species/or strains of microbes.

### 3.5. Health Benefits in Animals

*PCP* (Penthoraceae) traditionally has been used in China for the treatment of liver-related problems (infectious hepatitis, cholecystitis, jaundice, infectious hepatitis, edema, and antidrunk hangover). PCP showed hepatoprotective effects in broiler and other animals by decreasing liver injuries via various mechanisms. In ethanol-induced liver injury mice model, aqueous extract of PCP shows protective effect via decreasing CYP2E1-mediated oxidative stress and boosting oxidant defense mechanisms through activation of the Nrf2/HO-1 pathway. PCP also reported for correction of heat-related problems, diuresis, circulation activation, and protection of the spleen and liver [62]. PCP is involved in the reversion of jaundice and viral hepatitis and further corrected the serum indices of various biochemicals (insulin, triglycerides, TC, LDL-C, and HbA1c) and oral glucose tolerance test [63]. The liver injuries by carbon tetrachloride (CCl4) can be improved by treatment of PCP. Malondialdehyde levels are reduced, glutathione (GSH) is restored, superoxide dismutase (SOD) and catalase (CAT) activities are improved, hepatic cytochrome P450 2E1 (CYP2E1) is prevented from degrading, and nuclear factor erythroid 2-related factor 2 (Nrf2) and its target proteins are improved in CCl4 treated mice [28]. In many studies, PCP has been shown to lessen inflammation, liver fibrosis, viral infection, the balance of important liver enzymes, the activation of hepatic stellate cells, and hepatic virus DNA replication. Animal testing, however, reveals neither toxicity nor negative effects [60].

In dogs, acute liver injury due to CCl4 and oxidative stress due to which free radicals are produce that generate free radicals can directly cause lipid peroxidation in the cell membrane and cause cell membrane destruction. Hepatocytes can experience oxidative stress, degeneration, hepatocellular injury, and necrosis by the production of ALI. The ALI has shown improvement in vacuolar inflammatory lesions in liver tissues when treated with PCPE, restored glutathione peroxidase, enhanced activity of superoxide dismutase, and significantly lowers the serum levels of nitric oxide and malondialdehyde. By using PCPE, inflammatory factors were downregulated and anti-inflammatory factors were upregulated. In dogs with ALI, PCPE therapy decreased the levels of MEKK4, MKK3, p38MAPK, MSK1, and NF-B and increased the levels of IkB mRNA [45]. Similarly, PCP blocked the expression of cytochrome P450 2E1 and production of intracellular reactive oxygen species and decreases liver fat accumulation and oxidative damage. It upregulates the nucleus factor E2-related factor 2 (Nrf2) and downregulates the expression of Kelch-like ECH-associated protein 1 (Keep 1). PCP upregulates autophagy signaling pathways [1, 60].

**Figure 3:**
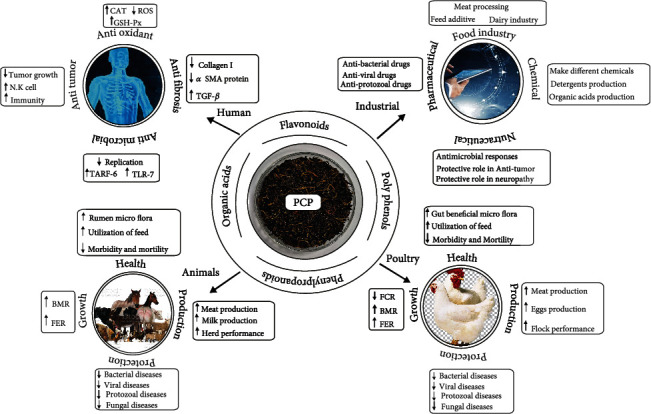
Schematic diagram illustrating the therapeutic effects and underlying mechanism of action, which showed the beneficial effect on poultry and animal health and production, highlighting the industrial application of PCP.

Administration of PCP extract in broiler chicken in aflatoxin B1-induced liver damage and oxidative stress can be reduced significantly. Aflatoxin poses a great threat to the poultry industry. PCP enhances growth performance, immunoglobulin level, and oxidative capability and reversing oxidative stress and pathological lesions in the liver; PCP administered to AFB1-affected birds lessens the negative effects; also, apoptosis was reversed [25, 26].  Figure 3 is a schematic diagram illustrating the therapeutic effects and underlying mechanism of action PCP in different animals.  Table 1 summarizes the studies with PCP and biological effects in different animals.

### 3.6. Immunomodulatory Effects

Traditional Chinese medicines are recognized as balancing methodology to modern medication and most promising and a safe alternative therapeutic alongside proven the immunomodulation agents in clinical practice [64, 65]. Immunomodulators promote the defense mechanism against the pathogens including viruses, thus supporting the immunity and homeostasis and could be an effective way to prevent the infections and pathogenesis. Several chronic diseases, allergies, viral infections, immune disorders, metabolic diseases, cancer, and inflammations are associated with immune system. Flavonoids, sterol compounds, polysaccharides, carotenoids, and terpenoids are important phytocompounds with well-known chemical structures and significant immunomodulating properties [66]. PCP contains the main types of components that are polyphenols, flavonoids, coumarins, lignans, organic acids, and sterols. It is proved that polysaccharides (galactose, arabinose, galacturonic acid, rhamnose, and glucose are polysaccharides) in PCP are involved in the immunoregulation of H_22_ tumor-bearing mice. In vitro research indicates that PCP-4 inhibits the growth of xenograft tumors by safeguarding immune organs, enhancing immune cell activity, and encouraging apoptosis [67]. PCP in common carp (Cyprinus carpio) improved the gut microbiota population and finally improved the intestinal immunity [44].

Gallic acid from PCPE is investigated for the immunomodulatory effects in immunosuppressed Swiss albino mice (cyclophosphamide and cisplatin). Gallic acid with different dosage (100, 200, and 400 mg/kg) regimens was orally administered for seven days. The results show that gallic acid could be used as an adjuvant immunosuppressive drug to decrease the adverse effects of immunosuppressive agents on the immune system [68, 69].

## 4. Conclusion and Future Directions

This updated review critically explained that PCP contains a wide range of medicinal important phytochemicals like flavonoids, phenylpropanoid, polyphenol, and organic acids. The bioactive component of PCP shows medicinal properties like antimicrobial, hepato, neuroprotection, anti-inflammatory, and strong immunoregulatory effects. Most of effects are mainly concerned with the regulation of apoptosis, mitochondrial, *AMPK*, autophagy, *TLR4*, *Keap1–Nrf2*, *NF-κB*, *p38-MAPK*, *IRT3-TGF-β*, and *Nrf2* signaling pathways. In poultry, it can be used as feed additive for health-promoting effects including liver tonic (detoxification of xenobiotics and repairing of liver), gut microflora for boosting immunity/or antimicrobial effect, and washing out of infectious microbes. In large animals, it protects different metabolic injuries improving gut health. In the future, it may be used in lactating animals for improving milk yield and activation of beneficial ruminal microbes' population. It is suggested that various industries (pharmaceutical, nutraceutical, food, and cosmetic) should increase the production capabilities due to variety of beneficial effects of PCP on animal health.

## Figures and Tables

**Table 1:** A summary of studies with PCP and biological effects on animals.

Study model/animal type	Dose and extract type	Results	Treatment and overall effect	Reference
Chicken	1, 2, and 3 g PCPE/kg feed	↑ growth ↑ immunoglobulin level ↓ oxidative stress ↓ Bax, Bak, caspase-9, caspase-3, and p53 ↓ pathological lesions in liver	Treatment of liver injury and oxidative stress	Nabi et al. [25, 26]
Chicken	5, 10, and 15 mL PCPC/kg feed	↓ Bax, Bak, cytochrome c, caspase-9, and caspase-3) ↑ NRF2 and HMOX1 ↓ apoptosis in the kidneys	Mitochondrial pathways in the kidneys	Tao et al. [45]
Dog	0.5, 1, and 1.5 mL PCP/dog herb mL/dog	↓ vacuolar inflammatory lesions in liver tissues ↓ IL-1*β*, IL-6, TNF-*α*, MEKK4, MKK3, p38MAPK, MSK1, and NF-*κ*B ↑ IL-10, IkB mRNA ↑ activity of superoxide dismutase	Hepatoprotective effect in dogs	Tao et al. [45]
HSCs LX-2 and HSC-T6 cells in rat	100 *μ*g/mL PCE	↓ collagen I ↓ *α*-SMA protein levels ↓ PI3K-Akt pathway ↑ TGF-*β*-Smad pathway	Liver fibrosis	Zhou et al. [15]
Female Kunming mice 20 ± 2 g and 6 to 8 week	100 and 300 *μ*g/mL purified polysaccharide fraction of leaves of PCP	↑ immunity, i.e., NK cells and lymphocytes ↓ tumor growth	Anticancer	Chen et al. [69]
Mice	PCE 166.6 ± 20.1 U/mL	↑ CAT and GSH-Px	Antioxidant	Yin et al. [16]
HUVEC cells/Mice	PCE 10-30 *μ*g/mL	↑ scavenging ROS ↑ Nrf2-antioxidant signaling pathway, ↓ oxidative-stress, ↓ pro- and cleaved-IL-1*β*	Treatment of cardiovascular diseases, Autophagy induction	Sun et al. [3–5]
Liver cells (L-02 cells cultured)	—	↑ expression of TNF-*α* and IL-6 at mRNA and protein levels ↓ mRNA and protein expression of Nrf2 and HO^−1^	Protect liver injury	Ding et al. [70]
Rat	PCE 545 mg/kg/da	↑ GSH-Px, SOD, and CAT ↓ levels of MDA	Diabetic treatment	Hu et al. [71]
Normal rat's liver cell (BRL-3A)	6.25–100 *μ*g/mL stem extract	↑ scavenging ROS ↓ liver enzymes directly ↑ antioxidant levels ↓ cellular apoptosis ↓ lipid peroxidation	Hepatoprotective activity	He et al. [12]
APP/PS1 mice	Thonningianin A (10 *μ*M) compound from PCP leaves, stems, or flowers	↑ in microglial cells triggered by A (1-42), NLRP3 inflammasome is degraded by autophagy ↓ neural destruction	Alzheimer's disease treatment	Zhou et al. [72]
Mice	PCP aqueous extracts 10.30 g/kg	↓ oxidative stress caused by CYP2E1 ↑ oxidant defense mechanisms *↑* HO^−1^ and Nrf2 pathway	Prevents ethanol-induced chronic liver damage	Cao et al. [62]
Rats	PC extract 150 and 300 mg/kg/day	↓ HbA1c, TG, and TC ↑ insulin	Antihyperglycemic effects	Suna [63]
Zebra fish	PCP polyphenols	*↑* Keap1–Nrf2	Neuro-protective	Sun et al. [73]
Zebra fish larvae	PCPE 25, 50, and 100 *μ*g/mL for 48 h	*↑* Keap1/Nrf2 ↓ mTOR/PI3K/Akt P2X7R blocking	Hepatoprotective via antioxidation and autophagy	Zhao et al. [46, 47]
HEK293T cells B16F10 cells	50, 100, or 200 *μ*g/mL Pc-EE for 24 h	↓ tyrosinase ↓ melanin ↓ LC3B	Antiapoptotic, antiaging, anti-inflammatory, and antimelanogenic properties	Jeong et al. [7, 8]
Rat	3 g/kg	*↑* cell proliferation inhibition *↑* scavenging ability	Antioxidant Antihepatocarcinoma	Lu et al. [74]
RAW264.7 cell with LPS	15, 30, 60, and 120 *μ*g/mL	*↓ NO*, *TNF-α*, and *IL-1β*	Anti-inflammatory	Lin et al. [30]
Rat	800 mg/kg	*↓ TG*, *TC*, *ALT*, and *AST*	Antilipogenesis	Yuan and Ou [75]
HepG2 cells	1, 10, and 100 *μ*M	*↑ AMPK/SRIT1* *↑ PPAR-α*	Antioxidant	Guo et al. [76]
Fish	5.2 to 6.1 mg/L	*↓ Sobs*, *ACE*, and *Chao* indices *↑ Bacteroides* *↓ Actinobacteriota* and *Fusobacteriota*	Anti-inflammatory	Ke et al. [44]

PCE: Penthorum chinense extracts; HSCs: hematopoietic stem cells; LX-2: human hepatic stellate cell line; PI3K-Akt: protein kinase B; TGF-*β*: transforming growth factor; *α*-SMA: *α*-smooth muscle actin; NK cells: natural killer cells; CAT: catalase; GSH-Px: glutathione peroxidase; ROS: reactive oxygen species; Nrf2: nuclear factor erythroid 2-related factor; COX-2: cyclooxygenase 2; IL-6: interleukin-6; IL-1*β*: interleukin-1*β*; NLRP3: Nod-like receptor protein 3; TNF-*α*: tumor necrosis factor-*α*; SOD: superoxide dismutases; MDA: malondialdehyde; CYP2E1: cytochrome P450 2E1; HbA1c: serum glycosylated hemoglobin A1C; TG: triglyceride; TC: total cholesterol; AMPK: AMP-activated protein kinase; TC: total cholesterol; TG: triglyceride; NO: nitric oxide.

## Data Availability

The data supporting this review paper are from previously reported studies and datasets, which have been cited.

## Abbreviations

ALP: Alkaline phosphatase
ALT: Alanine aminotransferase
AST: Aspartate transaminase
ATGL: Triglyceride lipase
CAT: Catalase
CCl4: Carbon tetrachloride
DPPH: 2,2-Diphenyl-1-picrylhydrazyl
GIT: Gastrointestinal tract
GSH: Glutathione
HG: High glucose
HUVEC: Human umbilical vein endothelial cells
IL: Interleukin
LDLR: Low-density lipoprotein receptor
LPS: Lipopolysaccharide
MRSA: Methicillin resistance S. aureus
MDA: Malonaldehyde
NLRP3: Nod-like receptor protein 3
PCP: Penthorum chinense Pursh
PCPC: Penthorum chinense Pursh compound
PCPE: Penthorum chinense Pursh extract
PCSK9: Proprotein convertase subtilisin/kexin type 9
ROS: Reactive oxygen species
TNF-*α*: Tumor necrosis factor-*α*
ThA: Thonningianin A (TA)
TCM: Traditional Chinese medicines
NO: Nitric oxide
VIPR1: Vasoactive intestinal polypeptide receptor 1.
